# Kidney transplant in diabetic patients: modalities, indications and results

**DOI:** 10.1186/1758-5996-1-2

**Published:** 2009-08-26

**Authors:** Érika B Rangel, João R de Sá, Cláudio S Melaragno, Adriano M Gonzalez, Marcelo M Linhares, Alcides Salzedas, José O Medina-Pestana

**Affiliations:** 1 Division of Nephrology, Universidade Federal de São Paulo, Brazil; 2 Division of Endocrinology, Universidade Federal de São Paulo, Brazil; 3 Departament of Sugery, Universidade Federal de São Paulo, Brazil

## Abstract

**Background:**

Diabetes is a disease of increasing worldwide prevalence and is the main cause of chronic renal failure. Type 1 diabetic patients with chronic renal failure have the following therapy options: kidney transplant from a living donor, pancreas after kidney transplant, simultaneous pancreas-kidney transplant, or awaiting a deceased donor kidney transplant. For type 2 diabetic patients, only kidney transplant from deceased or living donors are recommended. Patient survival after kidney transplant has been improving for all age ranges in comparison to the dialysis therapy. The main causes of mortality after transplant are cardiovascular and cerebrovascular events, infections and neoplasias. Five-year patient survival for type 2 diabetic patients is lower than the non-diabetics' because they are older and have higher body mass index on the occasion of the transplant and both pre- and posttransplant cardiovascular diseases prevalences. The increased postransplant cardiovascular mortality in these patients is attributed to the presence of well-known risk factors, such as insulin resistance, higher triglycerides values, lower HDL-cholesterol values, abnormalities in fibrinolysis and coagulation and endothelial dysfunction. In type 1 diabetic patients, simultaneous pancreas-kidney transplant is associated with lower prevalence of vascular diseases, including acute myocardial infarction, stroke and amputation in comparison to isolated kidney transplant and dialysis therapy.

**Conclusion:**

Type 1 and 2 diabetic patients present higher survival rates after transplant in comparison to the dialysis therapy, although the prevalence of cardiovascular events and infectious complications remain higher than in the general population.

## Introduction

Diabetes is a disease of increasing worldwide prevalence, with a perspective of 330 million people affected by 2030. In the United States of America, diabetes is the main cause of chronic kidney failure, corresponding to approximately 40% of the patients undergoing renal replacement therapy and outgrowing the number of cases of nephritis and systemic arterial hypertension (United States Renal Data System, 2004). Throughout the last four decades, it has been observed a change in the time interval from the diabetes diagnosis to the development of chronic kidney failure, which was shown to increase from 20 to 30 years [1]. In Brazil, it is estimated that there are 6 million diabetics being 5-10% of these type 1 diabetic patients and the others have type 2 diabetes. The worldwide incidence of type 1 diabetes is quite variable, but it is reported to be around 5-10% among those who are under 15 years old, and nearly 10% in Brazil [2]. The inicidence of type 2 diabetes has increased approximately by 52% from 1997 to 2003 in the United States of America [3].

Patients with type 1 diabetes and chronic kidney failure have the following therapy options, besides the dialysis treatment: kidney transplant from a living donor, pancreas transplant after kidney transplant, simultaneous pancreas-kidney transplant, or awaiting a deceased donor kidney transplant [4]. On the other hand, for type 2 diabetic patients is recommended kidney transplant from a deceased or a living donor.

### Isolated Kidney Transplant from a Living Donor for Type 1 Diabetic Patients

The awaiting time is shorter in this transplant modiality than in other ones because it takes just the time to prepare the living donor. Imunossuppressive regimen may be tailored according to the Human Leucocytes Antigens (HLA) compatibility. If there is is an identical donor, the dose of the immunosuppressors will be lower, which minimizes their side effects. This is the transplant modality that has the greatest life expectancy rate, around 18.3 years [4].

### Isolated Kidney Transplant from a Deceased Donor for type 1 Diabetic Patients

In Brazil, the organ allocation for kidney transplant from a deceased donor is determined by the HLA compatibility, resulting in a waiting time that may be over five or six years, which increases the secondary clinical complication events, either related to the diabetes or to the chronic kidney failure. The patient survival rate, although longer than in dialysis, is around 11.4 years [4].

On the other hand, it has recently been discussed if the comparison of the kidney survival can be done without a bias between simultaneous pancreas-kidney transplant and deceased donor kidney alone, inasmuch as there are donor, recipient and transplant differences that may interfere with the outcomes [5]. In this way, 5-year kidney graft survival was demonstrated to be similar between recipients of simultaneous pancreas-kidney transplant and kidney alone from pancreas donors (76.2% *vs *81.9%, respectively), whereas the kidney graft survival of the kidney alone from non-pancreas donors was significantly lower than the other two groups (64.3%). When controlling for recipient and transplant differences, it was observed a reduction of 50% in the risk of kidney graft loss in the group of kidney alone from pancreas donors in comparison to simultaneous pancreas-kidney transplant [5].

### Pancreas after Kidney Transplant (PAKT) for type 1 Diabetic Patients

Pancreas after kidney transplant (PAKT) modality has the shortest waiting time for pancreas transplant in Brazil, since many teams capture the pancreas, but do not perform the pancreas transplant surgical procedure, which results in a shorter waiting time in comparison to simultaneous pancreas-kidney transplant. The life expectancy rate is reported to be 17.2 years [4]. However, the greatest challenge is to monitor the pancreatic rejection, inasmuch as asynchronous pancreas rejection may occur.

Furthermore, PAKT indications are still controversial in the literature, but they may be certainly indicated for cases of severe asymptomatic hypoglycemia and ketoacidosis of difficult control [4]. Therefore, the probability of hypoglycemia greater than 5% and the probability of death from hypoglycemia greater than 9% justify the PAKT performance [4]. The PAKT is also indicated in cases when ketoacidosis becomes a frequent event (100.4 events for every 100 patients a year) [4].

In addition, it was recently demonstrated that the 1-year patient survival after PAKT was similar to the simultaneous pancreas-kidney transplant (98% *vs *95%, respectively), as well as the 1-year pancreas graft survival (95% *vs *90%, respectively) [6]. The immunosuppressive regimen included rabbit anti-thymocyte globulin (thymoglobulin), early steroid withdrawal and maintenance with tacrolimus and sirolimus or mycophenolate mofetil. Interestingly, the acute cellular rejection was present in only 2% of the groups described. However, further studies are necessary to evaluate the long-term pancreas graft survival rates after the PAKT.

### Simultaneous Pancreas-Kidney Transplant (SPKT) for type 1 Diabetic Patients

In type 1 diabetic patients under dialysis, the 5-year patient survival, even for young patients, ranges from 25 to 35%, increasing to 80% after the transplant [7]. In this way, the simultaneous pancreas-kidney transplant (SPKT) for type 1 diabetic patients is a therapy that increases patient survival. However, the type 1 diabetic patients who received a kidney transplant have a death risk of 4.3 and a higher cardiovascular morbidity rate, which is around 4.5, in comparison to the non-diabetic patients [8].

Confirming the transplant benefits with regards to the life expectancy of type 1 diabetic patients in comparison to the dialysis therapy, it was demonstrated that patients that are awaiting a deceased donor kidney transplant have an 8-year life expectancy as opposed to the patients that have 12.9-year, 21-year and 23-year life expectancies after the kidney transplant from deceased donors, the kidney transplant from living donors and the SPKT, respectively [9].

However, other authors reported a shorter life expectancy after the SPKT in comparison to the kidney transplant from living donors in type 1 diabetic patients [4]. It could be in part explained by the higher mortality rate in the first 90 days after the SPKT, once the surgical and infectious complications are more frequent [10,11].

Nowadays, the pancreas graft survival tends to be longer after the SPKT. There was shown an increase from 75% to 85% in the pancreas graft survival rates in the first year as compared to the other modalities of pancreas transplants, such as pancreas transplant alone and PAKT, which showed pancreas graft survival rates around 75% at 1-year [12]. After the SPKT, the secondary complications in type 1 diabetic patients are reduced and it is reported to occur clinical improvement in the carotid intima-media thickness [13], coronary atherosclerosis [14], diastolic dysfunction [15-17], left ventricle ejection fraction [15-17], peripheral neuropathy [18,19], cardiorespiratory reflexes [20], gastroparesy [21], as well as stabilization of the diabetic retinopathy [22,23].

### Transplant vs Dialysis for type 2 Diabetic Patients

Regarding patients with chronic kidney failure that are under renal replacement therapy, data collected from the USRDS (*United States Renal Data System*) from 1988 through 1996 indicated a 23% decrease in the mortality rates of the patients awaiting a deceased donor kidney transplant, which was also associated with a 30% decrease in the mortality rates of the patients submitted to kidney transplant [24]. Nowadays, one-thrid of the kidney transplant patients in the United States of America and also in Brazil has diabetes before the transplant. Despite the transplant benefit, the probability of a cardiovascular event after the transplant is higher in diabetic patients in comparison to the general population [24].

In the United States of America, the kidney transplants in type 2 diabetic patients are usually performed with deceased donors, while only 20% are performed with living donors [25]. It is also a reality in Brazil, inasmuch as the diabetic patients show lower probability of having living donors in the family, once there is a genetic predisposition for diabetes. According to Wolfe at al, the mortality after the kidney transplant, regardless of the age range (20-74 years old), is lower in comparison to the dialysis therapy [26]. Dialysis is a well-known risk factor for mortality because it contributes to the increased prevalence of cardiovascular events.

After the kidney transplant, the type 2 diabetic patients have 8 to 19 additional years in their life expectancy [26]. In addition, in American and non-American centers, there is a growing trend towards transplants in the elderly patients. The survival rates for either the patient or the kidney graft are directly correlated to the recipient's age and to the time of diabetes history, resulting in a reduction of 4 to 7% of the kidney graft survival rates (2002 United States Organ Procurement and Transplantation Network and the Scientific Registryof Transplant Recipients - OPTN/SRTR Annual Report). As opposed to these data, other authors demonstrated that in the type 2 diabetic patients, the 1-year, 3-year and 5-year kidney graft survival rates were similar to the non-diabetic patients (82.7% *vs *87.6%; 70.9% *vs *79%; 63% *vs *72.5%, respectively) [27]. However, a bias of this study is that there were significantly more living donors in the group of the diabetic patients in comparison to the non-diabetics (33.5% *vs *17.2%, respectively). In other study conducted with type 2 diabetic and non-diabetic patients, the patient survival rates regarding kidney grafts from deceased donors were 68% *vs *80% and from living donors were 81% *vs *90%, which suggests that the great differential seems to be the recipients' age, which has affected both the diabetic and the non-diabetic patients' survival rates [25]. Boucek et al also reported that the patient survival rates in 1-year and 5-year for type 2 diabetic patients were compatible to 85% and 69% respectively, and for the non-diabetic patients the rates were 84% and 74%, respectively, being the difference not statiscally significant. In addition, the 1-year and 5-year kidney graft survival rates censored for death were comparable between diabetics and non-diabetics (84% *vs *82% and 77% *vs *77%, repsctively) [28].

### Post-Transplant Clinical Evolution

The causes of kidney graft loss after the first year of transplant include chronic graft injury in 50% of the cases (30-40% chronic allograft dysfunction and 10-20% recurrence of primary disease), and the other 50% represent the death with a functioning kidney graft [29]. The physiopathology of chronic kidney allograft dysfunction includes mechanisms that involve either the alloantigens or the non-alloantigenens factors, such as donors' demographic data and events associated with the recipient, such as infection, hypertension, obesity, diabetes itself, dyslipidemia, nephrotoxicity due to calcineurin inhibitors and acute rejection episodes [29].

The main cause of mortality after the kidney transplant is cardiovascular diseases (37%), followed by the cerebrovascular diseases (7%), infections (20%), neoplasias (13%) and other causes (23%). The yearly cardiovascular mortality rate after the kidney transplant is 0.54% and for patients undergoing dialytic treatment it is over 9%, which is much higher in comparison to the general population (0.28%).

The risk factors for cardiovascular disease in diabetic patients are associated with insulin resistance, higher values of triglycerides and lower values of HDL-cholesterol, abnormalities in coagulation and fibrinolysis and endothelial dysfunction, so that the time of type 2 diabetes history has direct correlation to the mortality [30]. In this way, for each increment of 1% glycated hemoglobin in patients with 45 to 79 years old in a 6-year follow-up, there is an increase of 26% in cardiovascular events, either in men or in women [31]. As a matter of fact, even microvascular complications occurrence increase with the worsening of the glycated hemoglobin values [31].

More recent data demonstrated that the 5-year patient survival rate for type 2 diabetic patients was lower than the non-diabetics' (70 *vs *93%) [32]. When both groups were compared, it was shown that type 2 diabetic patients were older at the time of receiving the transplant, had higher body mass index (BMI), higher prevalence of pretransplant cardiovascular disease (48% *vs *16%), and higher incidence of posttransplant cardiovascular events (37% *vs *9%). In addition, the main cause of death after the transplant in type 2 diabetic patients included cardiovascular disease (61% *vs *26% for non-diabetics) [32]. Another interesting aspect was the observation that infection was the second cause of death after the transplant, and the diabetic patients also presented higher risk of mortality from infection causes in comparison to the non-diabetics [32].

Thus, in the non-diabetics, the 5-year patient survival rates after the transplant were mainly affected by the following factors: recipients' age and dialysis background *vs preemptive *transplant (transplant performed without previous dialysis therapy), while in the diabetic patients, the background of dialysis and cardiovascular disease have also influenced the patients' survival rates after the transplant [32].

Additionally, the incidence of cardiovascular events after the transplant in non-diabetic patients had a correlation to the traditional factors, while for the diabetic patients these cardiovascular events were associated with the presence of cardiovascular disease background [32].

For type 1 diabetic patients, it was demonstrated that 2-year patient survival rate after kidney transplant in comparison to the dialysis therapy was not different, though after the kidney transplant the lipid profile has improved (lower triglycerides and cholesterol values), but the average carotid intima-media thickness was not different, as opposed to the stabilization of the diabetic retinopathy in over 90% of the cases [33]. These findings point out to the benefits of the pancreas transplant for the type 1 diabetic patients. Moreover, the simultaneous *preemptive *pancreas-kidney transplant is associated with the longer survival rates, either for the patient or for the kidney graft [34].

In the longer follow-up, 5-year patient and kidney graft survival rates in type 1 diabetic patients that were submitted to SPKT and in the non-diabetics submitted to kidney transplant were 96% and 90%, and 85% and 75%, respectively [35]. Such differences may be explained by the fact that the the pancreas-kidney donors were younger and the kidney cold ischemia time was shorter [35]. In Brazil, the law determines that the pancreas-kidney donor is supposed to be less than 45 years old and without a positive family history of diabetes in the first degree relatives. In addition, the pancreas is not allocated by the HLA compatibility, which contributes to the lower cold ischemia times and consequently to lower rates of delayed graft function. In our center, the mean cold ischemia times for the kidney and the pancreas grafts are around 14 hours [11]. On the other hand, for the isolated kidney transplant, the cold ischemia time is approximately 24 hours, inasmuch as the kidney allocation is determined by the HLA compatibility.

After the SPKT, type 1 diabetic patients present a reduction in the left ventricular mass in the first year of the transplant, the glycated hemoglobin values also improve, as well as the BMI, the triglycerides values and either the systolic or the diastolic blood pressure control in comparison to the patients submitted to the isolated kidney transplant [13-17,36].

In addition, after 10 years the type 1 diabetic patients submitted to the SPKT in comparison to the isolated kidney transplant present lower prevalence of vascular diseases, including acute myocardial infarction, cerebrovascular events and peripheral arterial disease associated with amputation [37]. The 10-year survival of those patients was statiscally superior to the patients that only had an isolated kidney transplant (83% *vs *70%) [37], probably due to the lower progression of atherosclerosis [14]. These findings were also reinforced by the reduction of the carotid intima-media thickness after the SPKT, even though it does not become normal [13]. Regarding the peripheral vascular disease after the SPKT and isolated kidney transplant, an aggravation occurs in both groups, which is initially higher in the patients who have performed SPKT [38], probably due to the hyperinsulinemia, which may accelerate the atherosclerosis. It is also reported that amputation rates after the SPKT in type 1 diabetic patients are reported as 8.8 to 9.5% [39,40]. In type 2 diabetic patients under dialysis therapy, the yearly amputation rate is 6.1% and increases to 11.3% when the vascular disease is present on the occasion of kidney transplant [41]. So, in patients with pretransplant amputation, the risk of another amputation increases after the transplant.

Regarding autonomic neuropathy, after the SPKT, there is an improvement that starts after two years, which may justify the difference observed in the 10-year survival rates of the SPKT patients in comparison to the isolated kidney transplant patients, i.e., 80% *vs *20%, respectively [42]. However, more severe cases of autonomic neuropathy do not seem to present benefits from the transplant in relation to the patient survival [42].

Another discussed topic is if there is a room for glucose improvement after pancreas transplant in type 2 diabetic patients. In one study, the 5-year and 10-year survival rates for type 2 diabetic patients who were submitted to the SPKT were not different in comparison to the type 1 diabetic patients that were also submitted to the SPKT [43]. Yet, the number of patients evaluated was small and the definition criterion of the type 1 or 2 diabetes was based on C-peptide values lower or higher than 0.8 ng/mL, respectively, which is not sufficient to differentiate the diabetes types.

Another study has recently shown that 17% of the type 1 diabetic patients had hyperglycemia after the pancreas transplant, which has also occurred in 45% of the type 2 diabetic patients [44]. The risk factors for hyperglycemia after the pancreas transplant were associated with the type of transplant (SPKT *vs *PAKT and pancreas transplant alone), with type 2 diabetes *vs *type 1 diabetes history, with the pretansplant insulin dose and BMI, and with the acute rejection episodes occurrence [44]. By analyzing the BMI through the insulin dose prior to the transplant, it was observed that the BMI greater than 28 kg/m^2 ^and daily insulin dose greater than 70 U, regardless of the diabetes type (1 or 2), were the ones that had hyperglycemia in 80 to 90% of the times [44]. Thus, the indication for the pancreas transplant in those patients should be very cautious. The conclusion is that the type 1 diabetic patients that presented lower BMI and lower insulin dose in the pretransplant period were the ones that developed hyperglycemia less frequently, i.e., approximately in 11% [44].

After the transplant, the approach to the diabetic patients must be aggressive in relation to the systemic blood pressure control, weight control through diet and exercise, discontinuing smoking, proteinuria control, dyslipidemia control and hyperglycemia treatment, so that all the goals established prior to the transplant should be pursued in the posttransplant period [45]. Optimizing the conservative treatment measures and the approach with specific medications, such as Losartan, are associated with the decrease in the TGF-β expression in the kidney, which is a fibrosis marker, and with the reduction of proteinuria and consequently with the progression of chronic kidney allograft dysfunction [46]. Furthermore, the weight loss and the regular physical activity are associated with the improvement in the insulin sensitivity.

Glitazones are also associated with the reduction of insulin resistance and with the preservation of the beta cells mass. Besides, they have impact on the reduction of the cardiovascular risks and death rates of the patients with chronic kidney failure [47], further reducing the proteinuria and the systemic blood pressure. However, the posttransplant use of glitazones still requires more studies, especially in patients who are under corticosteroids treatment because the risk fracture due to osteoporosis may be elevated with these drugs.

## Conclusion

Type 1 and 2 diabetic patients show higher survival rates after transplant in comparison to the dialysis therapy, although the prevalence of cardiovascular events and infectious complications remain higher posttransplant than in the general population.

## Competing interests

The authors declare that they have no competing interests.

## Authors' contributions

EBR designed and wrote the paper, JRSA participated in the design of the study and read the paper, CSM participated in the design of the study, AMG participated in the design of the study, MML participated in the design of the study, AS participated in the design of the study, JOMP participated in the design of the study and coordination. All authors read and approved the final manuscript.
